# Machine Learning in Clinical Trials: A Primer with Applications to Neurology

**DOI:** 10.1007/s13311-023-01384-2

**Published:** 2023-05-30

**Authors:** Matthew I. Miller, Ludy C. Shih, Vijaya B. Kolachalama

**Affiliations:** 1 Department of Medicine, Boston University Chobanian & Avedisian School of Medicine, 72 E. Concord Street, Evans 636, Boston, MA 02118 USA; 2 Department of Neurology, Boston University Chobanian & Avedisian School of Medicine, Boston, MA 02118 USA; 3 Department of Computer Science and Faculty of Computing & Data Sciences, Boston University, Boston, MA 02115 USA

**Keywords:** Machine learning, Clinical trials, Neurology

## Abstract

We reviewed foundational concepts in artificial intelligence (AI) and machine learning (ML) and discussed ways in which these methodologies may be employed to enhance progress in clinical trials and research, with particular attention to applications in the design, conduct, and interpretation of clinical trials for neurologic diseases. We discussed ways in which ML may help to accelerate the pace of subject recruitment, provide realistic simulation of medical interventions, and enhance remote trial administration via novel digital biomarkers and therapeutics. Lastly, we provide a brief overview of the technical, administrative, and regulatory challenges that must be addressed as ML achieves greater integration into clinical trial workflows.

## Introduction

The term artificial intelligence (AI) refers to the use of computational methods to enable machines to perform tasks such as perception, reasoning, learning, and decision-making. Advances in the technology sector are fueling the development of novel forms of AI, which are rapidly driving progress across diverse domains such as facial recognition, financial strategy, and self-driving vehicles [1, 2]. The field of medicine is no exception, with AI methods increasingly being applied in healthcare research, from the laboratory to the bedside. In clinical trials, particularly, automated methods similarly carry great promise to alleviate many of the considerable difficulties associated with planning, completing, and analyzing the results of large scale trials. The challenges associated with traditional trials, from recruiting participants across diverse populations to the selection of feasible and appropriate eligibility criteria, make these interventions an ideal area for the application of emerging data science techniques.

In this article, we reviewed machine learning (ML) as a means of achieving AI and improving the practice of clinical research. We provided a basic introduction to key ML concepts for clinicians, surveyed general areas of application for ML in clinical trials, and then demonstrated how ML is being used to foster innovation in clinical research for neurologic diseases, specifically. We concluded with a discussion of technical challenges to automation in trials, highlighting potential obstacles that must be overcome to sustain innovation in the field.

## Background

### ML in Medicine: Why Now?

Efforts to standardize clinical care via advanced statistical models have their roots in the twentieth century [3, 4], when the advent of modern computers enabled researchers to begin simulating the process of differential diagnosis [3-8], recommending antibiotic regimens [9], and identifying medication effects [10]. Though these early initiatives fell short of making widespread impact [11], a number of factors have led to an unprecedented rate of progress in ML since the early 2010s.

Increased access to large quantities of electronic data (in medicine, most notably, publicly available datasets such as the UK Biobank [12] and the Cancer Genome Atlas [13]), advances in computer hardware (especially Graphics Processing Units [GPUs]), and the widespread availability of open source software [14] have created the necessary environment for AI to achieve significant gains. Furthermore, continued algorithmic developments have enabled machines to take on tasks of increasing complexity and nuance [15].

Recent advances in machine learning have been driven by the development of novel techniques that prevent overfitting [16-20], and improve training processes [21-23], leading to the maturation of the field. Modern deep learning frameworks such as convolutional neural networks (CNNs) have emerged as a powerful tool for computer vision tasks [24], enabling the extraction of salient visual features from natural and medical images without the need for manual intervention. In addition, the development of new “transformer” networks has revolutionized machine learning models’ ability to make context-aware predictions [25]. Overall, these advances have significantly improved the performance and versatility of deep learning in a range of applications. As a result, we have seen dramatic improvements in areas as diverse as speech recognition, driverless cars, and precision marketing of advertisements [26]. Medical innovation often follows directly from the progress made by software companies in non-clinical arenas [27], and healthcare researchers are increasingly using ML methods to augment clinician workflow, predict outcomes, and discover insights from medical datasets. From the accurate diagnosis and classification of skin cancer [28] to AI-based detection of diabetic retinopathy [29] to the potential for timely identification of Alzheimer’s disease using both neuroimaging and clinical data [30], medical ML is showing its prowess to provide high-value contributions to patients and clinicians.

### How Machines Learn: What Clinicians Should Know

While the notion of learning implies some measure of human-like agency, medical ML algorithms depend on the transformation of patient-derived data into numerical formats that can be processed by computer systems. For instance, computed tomography (CT) scans can be understood as matrices of pixel intensities, and vital sign measurements may be translated into lists or vectors of discrete measurements. If an investigator can derive numerical quantities from a given data source, then the possibilities for which modalities can be used as input to an ML strategy are nearly limitless.

With the data thus translated, ML models act according to principles encoded within their architecture. Supervised learning models, as an example, are traditionally composed of models that can be trained by minimizing an error, via a loss function, between their predictions and known quantities within a dataset that are typically provided by a human labeler [1, 2]. The loss function guides the model by adjusting its underlying mathematical structure (i.e., the parameters that govern the mappings from inputs to outputs) [31] so that the model can ultimately provide as output either a probabilistic estimate of a data point belonging to a certain category (in the case of classification tasks) or direct estimates of a continuous measurement (in the case of regression tasks). Nevertheless, the traditional paradigm of minimizing loss with human-supplied labels for prediction is increasingly in flux. Self-supervised learning models are coaxed to identify common patterns in data by being trained to associate samples with certain characteristics, such as those from the same source [32] (e.g., serial ECGs from a single patient). These models undergo “pretext” training to learn these associations without requiring explicit supervision and can then be repurposed for other tasks down the line, such as prediction. Reinforcement learning (RL) models, on the other hand, respond to “rewards,” which direct the model into adjusting its parameters such that it increases its probability of performing certain actions [33] (e.g., making appropriate decisions in response to sepsis in intensive care settings) [34]. Additionally, generative models produce novel data products from either structured inputs that are then enhanced in some way (e.g., production of high-resolution radiologic scans from low-resolution analogs) [35] or even from simply statistical noise [36].

While different ML algorithms carry their own sets of advantages and disadvantages, the choice of which to use may depend on the task of interest, the available data, access to proper computing hardware, and the investigator’s desire to elucidate mechanistic insights (i.e., interpretability) from the model. As an example, CNNs perform excellently in determining diagnoses from radiologic images. However, such models often contain millions of parameters, and when run on standard “central processing units” (CPUs), they are prohibitively slow to train and develop in iterative fashion. Specialized hardware, such as GPUs, are often needed to accelerate the pace of computation to a tractable timeline [37], but may not be as easily accessible in many environments. Logistic regression, on the other hand, may require little more than a desktop computer while yielding mathematical coefficients that can be intuitively interpreted in the context of the underlying data. Furthermore, complex models and ever-increasing amounts of data do not necessarily translate to higher performance. Simple data distributions (e.g., finding a best-fit line in a unidimensional scatterplot) do not require complex model architectures for adequate solutions to be discovered; indeed, in certain instances, simpler models may be found to perform near-equivalently to complex ones after comparison [38].

**Fig. 1:**
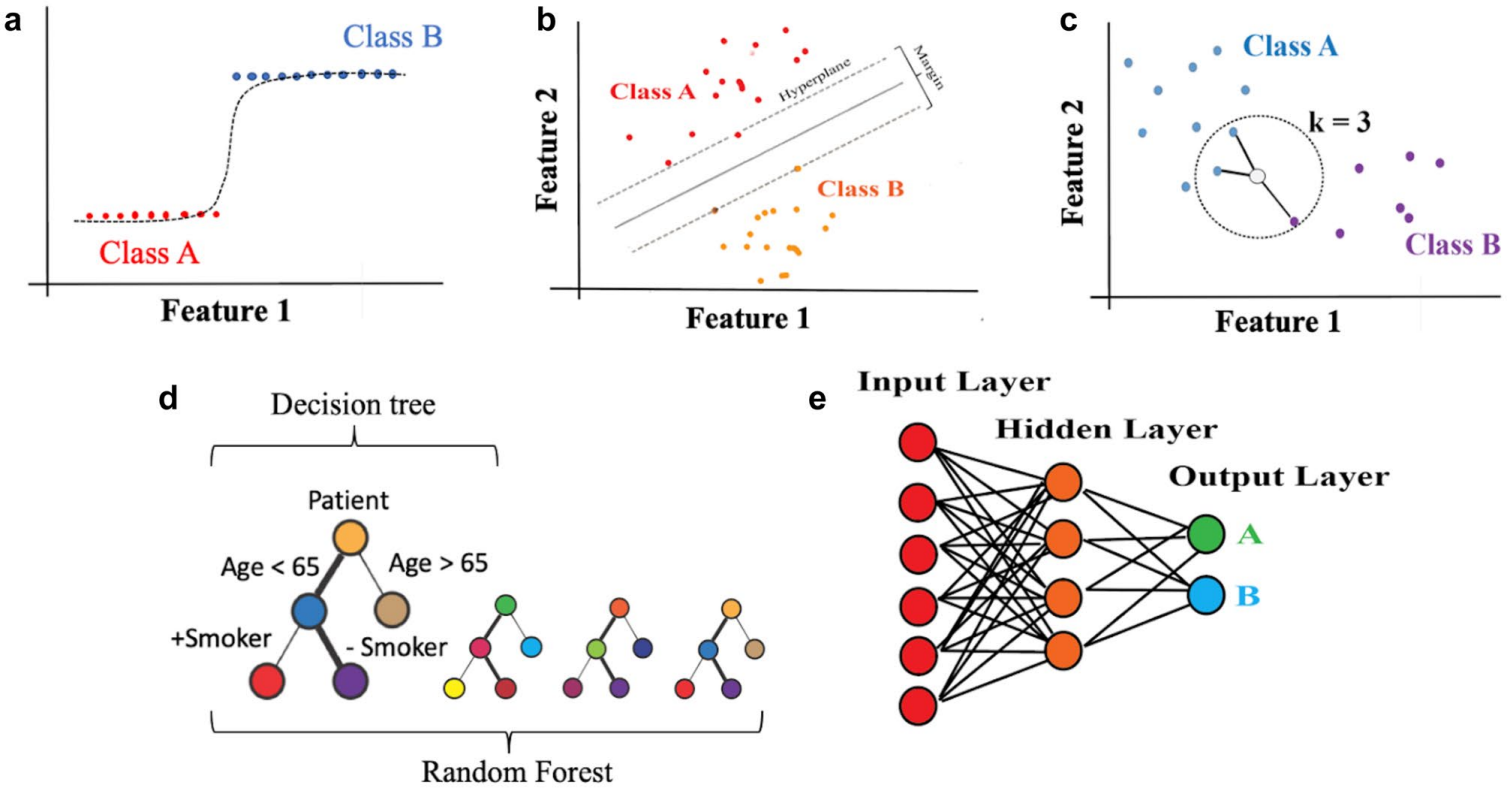
Graphical illustration of machine learning algorithms. Schemata of several exemplary ML algorithms are demonstrated. **a** Logistic regression; **b** support vector machine (SVM); **c** K-nearest neighbors (KNN); **d** decision trees and random forests (RF); **e** neural network (specifically, a multilayer perceptron/MLP)

Lastly, the performance of medical ML models can be assessed according to a variety of metrics, depending on the specific use cases. In the case of diagnostic or prognostic classification tasks, it is often standard to report area under the receiver operating characteristic curve (AUROC), obtained by plotting true positive rate versus false positive rate at differing probability thresholds when comparing predictions versus observation [39]. Area under the precision-recall curve (AUPR) (obtained from plotting positive predictive value versus sensitivity) may also be reported, as AUROC may overestimate performance in the case of highly imbalanced datasets [40]. A variety of specialized metrics for tasks such as segmentation (e.g., dice coefficient and intersection-over-union) [41], image generation (e.g., structural similarity) [42], and other tasks may also be deployed depending on the use case. Conversely, in regression for continuous quantities, standard metrics such as the mean squared error (MSE) between predicted and observed values may also be used [43]. Regardless of the specific measure employed, however, it is also imperative that ML models be judged in terms of traditional criteria (e.g., sensitivity, specificity, accuracy) in order to fully contextualize their impact on patient care prior to deployment. An overview of essential ML terminology along with definitions is provided in Table 1. Examples of widely used ML algorithms are illustrated in Fig. 1 and further elaborated in Table 2.

**Table 1:** Basic machine learning nomenclature

**Machine learning (ML):** The study of statistical models with the capacity to improve their predictive performance with exposure to data **Classification:** The task of using ML models to predict categorical labels from data e.g., predicting a diagnosis of Alzheimer’s disease from an MRI scan **Regression:** The task of using ML models to predict continuous labels from data e.g., predicting a neurocognitive test score from an MRI scan **Loss function**: A metric that quantifies an ML model’s prediction error. Over the course of training, the model “learns” to improve its predictions by minimizing the loss function **Training set:** The dataset of examples that an ML model uses to minimize its loss function. Therefore, this is the set of all observations with which the model is “trained” to detect patterns in data e.g., a retrospective dataset of case and control patients was used to train a model that diagnoses Parkinson’s disease **Testing set:** The dataset to which a fully trained ML model is applied. This dataset is used to gauge the ability of the ML model to function in making real-world predictions e.g., a prospective patient population in which a newly trained model will be used to diagnose Parkinson’s disease **Supervised learning:** A subtype of ML in which models learn to make predictions by minimizing the error between their predictions and a set of predetermined outcomes (otherwise known as labels) e.g., teaching a model to diagnose ocular palsies by exposing it to a training set consisting of pre-labeled videos of cranial nerve examinations **Unsupervised learning:** A subtype of ML in which models learn to infer patterns without being guided by predetermined labels. It does not require extensive manual labeling of data by human workers e.g., hypothesizing new subtypes of nonconvulsive status epilepticus from unlabeled electroencephalogram recordings **Semi-supervised learning:** A hybrid approach spanning supervised and unsupervised learning. With this approach, an ML model learns to make predictions on large quantities of unlabeled data using a smaller labeled dataset as a “guide” e.g., teaching a computer vision model to highlight areas of all areas of hemorrhage in a head CT when only several axial slices have been annotated by a neuroradiologist

**Table 2:** Examples of machine learning algorithms

**Logistic regression:** Often used to quantify effect sizes in traditional statistics, logistic regression (Fig. 1a) may be used as an ML model by assigning each feature of a dataset to a specific parameter and then tuning these on a training set. Additional adjustments to the model design (i.e., parameter regularization) may be used to decrease the model’s reliance on any singular feature and augment performance [44]. **Support vector machines (SVM):** SVMs (Fig. 1b) learn to set a decision boundary (“hyperplane”) that maximizes the separation (“margin”) between different sets of observations in the data space [45]. Decision boundaries may be adjusted to be nonlinear boundaries through specialized methods [46]. The relative simplicity of an SVM’s decision often makes this model a good choice for avoiding overfitting in complex datasets (e.g., longitudinal neuroimaging data such as fMRI) [47]. **K-nearest neighbors (KNN):** The KNN algorithm (Fig. 1c) predicts the outcome from a set of input features from the *k* most similar points, where k is a small integer chosen by the investigator [48]. In practice, KNN is often used as a data-informed strategy for imputing missing values in a dataset. E.g., in longitudinal cohorts, KNN can be used to estimate missing variables (e.g. neurocognitive test scores [49]) by training subjects for whom full data is available **Decision trees:** Decision trees (Fig. 1d) are essentially flow charts that can be used to predict outcomes based on branching logic. Given input feature values, the model undertakes a series of binary decisions to reach the proper outcome. Over the course of training, the model learns to navigate each branch point with increasing accuracy [50]. These models are best used when a high degree of interpretability is sought, but their performance may suffer relative to more complex modeling strategies **Random forests (RF):** RFs (Fig. 1d) combine many randomly generated decision trees to provide an overall prediction. The overall final prediction is the result of averaging the results from individual trees in the forest through procedures such as majority voting or arithmetic averaging [51]. So-called “boosting” algorithms [52] are often used in RFs to generate successive trees that minimize the errors of earlier trees and provide improved performance. Boosted RFs are often a good benchmark for non-neural network performance, with growing deployment in academic studies [53]. **Neural networks (deep learning):** Neural networks (Fig. 1e) use chains of mathematical functions (or “layers”) to make predictions. When many such layers are connected, the network is deep. Deep learning is a massive field that powers most of modern ML, and new “architectures” for networks are constantly emerging. However, we may note some crucial categories as follows: ** • Multilayer perceptrons (MLP):** These networks consist of an input layer of feature values that undergo further calculations in “hidden layers” before they are used to make a final prediction [54]. Such relatively simple networks may be used as standalone models or as parts of larger deep learning models ** • Convolutional neural networks (CNN):** CNNs use a series of filters that distill data patterns to their most essential properties. This is particularly useful in computer vision, where complex patterns of pixels (e.g., abscesses and organ boundaries) must be learned by building them up from simpler shapes (e.g. lines and edges) [55]. As such, these networks are best-use standards for tasks such as visual diagnosis and segmentation of critical structures in radiologic scans ** • Recurrent neural networks (RNN):** RNNs process sequential data using looped calculations that allow the network to form a “memory” of its previous processing behavior. This is highly useful for linguistic data and time series such as electrocardiograms [56] or electroencephelograms ** • Transformers:** Transformers are relatively new neural network models that differentially weight pieces of their input data using a concept known as “attention” [25]. This allows the appearance of contextual awareness in a model, such as in language tasks [58] (e.g., differing importance of different parts of a sentence) or computer vision [58] (e.g., differing importance of different areas of an image)	

**Learning point 1:** Machine learning frameworks have the potential to accelerate the timeline of clinical trials by facilitating patient selection via mining electronic health records.

## AI and Clinical Trials

### What Can be Gained?

Despite their successes, modern clinical trials remain difficult for research teams to bring to completion. Remarkably, unsuccessful trials remain the norm rather than exception due to myriad difficulties in identifying, enrolling, and providing treatment to patients within RCTs. Indeed, it has been estimated that only 12% of drug development programs achieve clinical trial success from phase 1 to launch [59]. While lack of clinical efficacy makes up a large component of the failures, many clinical studies fall short of recruitment goals and timelines due to factors such as low patient participation in clinical research and overly stringent inclusion criteria [60].

In what ways, then, can ML technologies help to alleviate these difficulties and advance new generations of clinical research? Here, we review several key areas in which such progress is already being demonstrated. We begin by discussing the power of natural language processing (NLP) approaches for sorting through large administrative databases and easing the work of identifying and screening potential participants. We next turn our attention to emerging methods for ML-based simulation of treatment interventions, which may one day challenge the supremacy of centralized, prospective studies. Lastly, we examine the possibility of medical software whose goal is not to support existing treatments but rather to act as the treatment in and of itself. These “digital therapeutics” require a rethinking of both the nature of medical therapy as well as the regulatory processes that govern the development and approval of drugs and devices.

### Clinical Trial Execution: Patient Recruitment and Eligibility Criteria

A uniform problem across industry-, foundation-, and federally- funded clinical trials is their significant financial costs and lengthy timelines. Recent surveys of phase 3 studies, for instance, have demonstrated median durations of more than 700 days between the initial planning of a study and its initiation [59], while the expense of recruiting patients meeting eligibility criteria consumes ~ 1 billion US dollars in annual research spending and up to 30% of development timelines [61]. Indeed, identification of study participants rather than the conduct of the trial itself currently accounts for some of greatest timeline delays. Furthermore, despite efforts to incentivize clinical trial sites to shorten recruiting timelines, identifying interested participants, adequately providing informed consent, and then conducting a medical history, physical examination, laboratory, and other diagnostic studies to assess eligibility criteria is often a laborious process requiring intensive review by research staff.

 Moreover, the dramatic increase in the availability of electronic health records (EHR) due to advances in information technology [62] has complicated the task of examining available data for identifying and pre-screening potential research participants. Ostensibly, the growth of health records has created both challenges and opportunities [63]. The International Classification of Disease (ICD) diagnostic codes used worldwide for clinical billing, for instance, could potentially be used to identify patients who have the condition of interest. However, diagnostic codes may also be misapplied by treating clinicians [64, 65], reflecting outdated or suspected but unconfirmed diagnoses. This inconsistency within EHRs  not only complicates efforts for maintaining an accurate clinical record but also affects the ability of research staff to leverage large databases to accurately pre-screen for clinical trials. Automated methods for maintaining an accurate medical history could be a particularly useful innovation.

Given these challenges, ML techniques capable of automatically screening the EHR from prospective participants are beginning to reshape the recruitment landscape in clinical trials. These advances are predominantly driven by NLP. Though a fuller discussion of ML-driven language processing and its applications in medicine may be found beyond this paper [66], NLP is now tapping into an ability to use large amounts of “unstructured” text data, such as that used in clinical notes, whereas previous generations of ML models may have required more strictly formatted data inputs. Text sources such as radiology reports and physical examination summaries may be “featurized” in a variety of formats, for instance, by scoring each document by the number of occurrences of unique words. More advanced deep learning-based methods such as large language models (i.e., BERT [67], GPT [68]) are being developed to accurately learn numerical encodings of individual words based on sentence context, thus endowing the next generation of neural networks with an ability to represent nuanced meaning in text.

NLP approaches are already being employed to derive insights from unstructured text data in clinical trials. IBM’s Watson supercomputer, for instance, has been shown in recent work to improve the efficiency of patient-trial matching, increasing monthly enrollment in clinical breast cancer trials by 80% using a combination of administrative patient records and eligibility criteria from ClinicalTrials.gov [69]. Similar performance has been shown in lung cancer, as well, where Watson recently achieved 91.6% accuracy in matching eligible patients to appropriate trials [70]. Remarkably, Watson achieved such performance by matching > 7000 separate patient attributes (including histologic reports, demographics, medical/surgical history, and genomics) with > 11,000 eligibility criteria across ten phase I–III trials. With an average runtime of 15.5 s per patient, the automated approach balanced remarkable accuracy with unprecedented speed, thus hinting at the possibility of greatly reduced timelines for patient recruitment.

Automated NLP tools for study recruitment are also being used directly by patients and clinicians, as certain research groups have begun to produce tools capable of translating simple queries into computer code which can be cross-referenced with online databases of study eligibility. Researchers at Columbia University, for instance, have developed open-source tools [71] to automatically match patients with studies on ClinicalTrials.gov. Enabling non-technical usage of NLP algorithms through online search tools has the potential to streamline the tedious process of determining one’s eligibility and may also democratize the usage of AI for key stakeholders. Similarly, several groups have demonstrated the viability of integrating NLP algorithms into the EHR platforms used by healthcare providers in routine care. By correlating the information contained within a patient’s medical record to databases of ongoing clinical trials, it is possible to create automated “alert systems” that flag a patient’s eligibility for participation in trials of interest [72, 73]. 

Work in ML-based simulation methods has also suggested ways in which eligibility criteria themselves may be adjusted to streamline patient enrollment for clinical trials. A recent study by Liu and colleagues ran thousands of simulations using published eligibility criteria from a database of > 60,000 patients participating in drug trials for advanced non-small cell lung cancers [60]. In order to elucidate the influence of individual eligibility criteria on trial outcomes, the authors adapted a statistical technique developed to quantify the influence of individual features on ML model predictions [74]. By systematically identifying the importance of each criterion, they were able to identify a core set of “data-driven” conditions that increased the number of eligible patients while minimally affecting the observed hazard ratios. Work such as this carries broad importance for clinical trial research by automatically highlighting criteria that study organizers can relax conditions for patient participation. Less stringent criteria will not only help to lower barriers to study recruitment but are also likely to increase the external validity of clinical studies given that poorly designed exclusion criteria may result in systematic biases within experimental populations.

Lastly, in an age of increasing awareness of healthcare inequality, ML methods for patient recruitment may be applied to alleviate racial disparities in clinical trials. Notably, it has been estimated that nearly 90% of participants in these studies are White [75], while historical surveys of clinical trials show that they are poorly representative of women, ethnic minorities, and patients outside of relatively wealthy regions such as North America or Western Europe [76, 77]. There is little doubt that drug and medical device development poses the risk of further alienating disadvantaged patient populations when ML-based methods used to validate them in clinical trials rely on data from non-representative groups [78, 79]. The generalizability gap, however, may in part be alleviated by automated methods for improving enrollment of historically underserved groups. Zhang and colleagues, for instance, have demonstrated the usage of ML classifiers to explicitly match pregnant women and persons living with HIV to oncology trials from ClinicalTrials.gov [80]. Health systems may also use enhanced screening capacity for trial eligibility to match patients from excluded groups to ongoing studies, either by NLP methods that explicitly take into account patient identities or from the types of data-driven eligibility expansions proposed by Liu and colleagues [81, 82]. Electronic phenotyping of disease characteristics rather than demographic factors may also identify which patients are most appropriate for enrollment on the basis of their physical health, though certain clinical phenotypes (e.g., poor pulmonary function and high BMI) may retain confounding relationships with race, ethnicity, class, and gender [83]. To enhance diversity in clinical trials, a promising strategy is to use ML to identify clinical sites that may benefit from focused resources aimed at training and recruiting investigative site personnel from underrepresented minority groups. These efforts can lead to a greater representation of diverse participants in clinical trials, underscoring the importance of prioritizing such initiatives.

**Learning point 2:** Machine learning techniques may help improve the efficiency of clinical trials by increasing the ease of recruiting research participants.

**Learning point 3:** Natural language processing techniques can help identify eligibility criteria from large quantities of electronic health records and then automatically connect an individual to ongoing studies. Simulation work in this area has also shown ways in which to relax overly stringent eligibility criteria without impacting study outcomes.

**Learning point 4:** Natural language processing techniques can identify participants from large databases and may help alleviate racial inequities in clinical trials.

### Going Beyond In-Person Trials: ML and Simulation

Given the time and expense associated with completing clinical trials, many investigators have turned their attention to alternative study designs for validating new therapies and diagnostics. With the increasing availability of large-scale health databases, novel strategies are now emerging to identify effective interventions for patients without the need to organize prospective trials. In addition, regulatory bodies are increasingly recognizing the value of such real-world evidence (RWE) as complementary to clinical trial-based evidence to support substantiation of a drug’s efficacy [84]. Nevertheless, ML models are subject to the same systematic issues in data collection that plague traditional statistical analyses, such as confounding, selection bias, and inconsistent data quality [85-87]. Therefore, without carefully controlled randomization, in what ways might a new generation of predictive algorithms enable the completion of simulated clinical trials to robustly compare healthcare interventions? Could ML spur the development of a new generation of virtual or simulated trials still capable of producing trustworthy results?

Already, there is widespread interest in using external datasets to augment the statistical power of traditional clinical studies, especially in rare diseases where parallel-arm, placebo-controlled studies may be limited by the number of trial participants available [88-90], including significant support from regulatory bodies in the USA, Canada, and Europe [91]. ML technologies such as NLP may help to advance these efforts by identifying cohorts in retrospective datasets who match the eligibility criteria of patients being treated in target trials [92]. Though additional efforts are likely required to ensure the comparability between the live and simulated study groups [93], synthetic cohorts may help to strengthen inferences in clinical studies where control groups cannot feasibly be recruited due to trial logistics for a low number of participants, such as in rare diseases [94].

Promising results are also being reported at the nexus of ML and causal inference (CI), a subfield of statistics dedicated to the identification of cause and effect in observational data [85, 86]. The fundamental challenge of CI is to quantify the difference between two separate outcomes: one that was observed (i.e., factual) and one that was not observed (i.e., counterfactual). Such a hypothetical inference may be estimated by scoring the likelihood of an individual receiving treatment (the so-called propensity score [95]) and then comparing clinical outcomes between similarly scored groups of treated and control patients [96]. Yet while such matching strategies have been shown to recapitulate the results of RCTs from observational data [97], calculating propensity scores by traditional methods may become difficult as the number of clinical variables collected from each patient becomes large [98]. Thus, ML models may also be used to derive enhanced estimates of these metrics by learning to predict treatment assignments from large quantities of data. Deep learning may even be used to provide simulated patients with propensity score matching, thus enabling the expansion of observational datasets with semisynthetic comparison groups to estimate treatment effects [99].

Lastly, a variety of research groups have now shown the capability of neural networks to learn shared patterns of characteristics (i.e., representations) between subjects receiving different forms of treatment [100]. After optimizing the identification of commonalities between patients in different treatment arms, these networks may then be used to quantify the effects of different interventions by simulating clinical outcomes in the presence or absence of a given treatment [100-102]. Such approaches essentially create “digital twins,” or virtual avatars, of individual patients that may then safely be subjected to experimental therapeutics [103-105]. Still early in development, these systems may one day provide accurate, unbiased estimates of treatment effects from readily available retrospective datasets. Though time will tell, the ability to draw causal inferences by ML-driven simulations could help prioritize or modify the design of interventional RCTs by simulating the prior probability of success of an intervention without the need to even enroll a single patient.

**Learning point 5:** Combining machine learning with causal inference techniques can help investigators to assess cause-and-effect from observational data. This synergy can facilitate investigators in assessing the impacts of medical treatments without the need to organize large prospective studies.

### Innovating Trial Design: Remote Monitoring, Digital Biomarkers, and Therapeutic Software

ML may also be used to improve the efficiency of clinical trials by alleviating many of the burdens associated with traditional, centralized study designs. In the era of COVID-19, for instance, researchers have discovered that many of the tasks previously required of patients may be completed via remote telemedicine, including the processes of obtaining informed consent [106], administering experimental drugs [107], and completing study questionnaires [72]. Given that factors such as severe illness and travel burden may contribute to patient dropout in clinical trials, remotely conducted trial visits may help investigators to retain study participants and increase the odds of a successful trial. However, when study visits are not being overseen in the clinic by research personnel, automated methods may also be able to provide quality control and ease administrative tasks.

There are myriad ways in which ML can aid remote trial administration. The US Food and Drug Administration (FDA), for instance, recently developed a mobile application (*MyStudies*) to support informed consent during the coronavirus pandemic [108]; the security of such systems may conceivably be improved by training image classification algorithms to confirm the veracity of patient signatures. Similar approaches have been adopted to confirm adherence to medication regimens in patient populations such as those experiencing mental illness or substance use disorders. As an example, AiCure, an analytics company specializing in remote clinical trial support, has employed facial recognition technology to confirm whether patients with opioid addiction are adhering to assigned medication regimens [109]. Tokyo-based Otsuka Pharmaceuticals has also piloted the usage of ingestible sensors in order to monitor the ingestion of antipsychotic drugs in patients with schizophrenia [110].

Remote monitoring of factors such as vital signs and blood chemistry could also provide early detection of adverse events in clinical trials by automatically flagging dangerous fluctuations in a participant’s state of health [72]. Given the power of ML systems to detect anomalies in continuous signals [111], software programs that learn a patient’s unique physiologic patterns from wearable or implantable sensors may lead the way for personalized warning systems during experimental drug trials. Additionally, ML models can learn entirely new patterns from standardly collected data, giving rise to a new generation of digital biomarkers [112, 113], to monitor treatment responses. Automated systems may learn to detect these biomarkers from a singular data source (e.g., electrocardiogram) or from combinations of multiple modalities (e.g., pulse oximetry, skin conductance, and blood glucose) to maximize the amount of information used for decision-making. In addition, physiological signals or digital markers of real-world function, such as the use of wearable sensors to quantify mobility, may ultimately serve as clinical efficacy outcomes themselves [114, 115]. Regardless, ML may enhance the ability of the clinicians to ensure the safety of a clinical trial participant who is taking part from home and is not in the clinic.

Finally, evidence is emerging that new digital technologies may act as treatments themselves rather than simply supporting the development of traditional drugs and devices. Such “digital therapeutics” [116], including prescription video games and mobile applications, are now in the pipeline to treat conditions as diverse as ADHD, addiction, psychosis, and multiple sclerosis (MS) [117]. Though not all digital therapeutics use ML algorithms to carry out treatment, there is increasing consensus that ML technology will be required for these products to achieve future standards of precision medicine [118], and developers of these technologies are actively partnering with AI researchers to personalize and improve their delivery [119]. FDA approval and the granting of specialized “pre-certification” pathways for developers of digital therapeutics are encouraging many companies to break into this space, including both traditional pharmaceutical firms and software startups [120]. The digital revolution, with ML at its core, may bring new players to medical innovation, inevitably bringing changes to the clinical trial landscape as they seek to validate entirely novel concepts of disease therapies.

**Learning point 6**: Machine learning may help to alleviate obstacles to remote participation in clinical trials by enabling more effective offsite monitoring of patient well-being and adherence to medication regimens. Algorithms can help to make sense of standard data streams (e.g., vital signs) or may be trained to derive novel digital biomarkers that can provide improved prediction for outcomes of interest. Machine learning may also accelerate development of digital therapeutics, in which software itself acts as a treatment for disease.

## Case Study of AI in Clinical Trials: Applications to Neurology

The great degree of variability in the presenting symptoms of neurologic disease often renders the identification of eligible patients, monitoring of progress, and evaluation of treatment endpoints in clinical trials difficult, even when performed by experienced clinicians [121]. Indeed, the complexity of neurologic disease is a likely contributor to low rates of success in clinical trials relative to other domains of medicine [122], and projected shortages in the neurologist workforce over coming decades [123, 124] threaten to exacerbate this trend. In this context, AI methodologies offer considerable benefits for clinical trials in neurology moving forward.

With respect to eligibility and recruitment, NLP offers promise across a range of clinical trials encompassing both acute and chronic conditions. In vascular neurology, for instance, NLP has been demonstrated to successfully characterize ischemic stroke from neuroradiology reports, automatically identifying TOAST [125] subtypes [126], location and acuity [127], and critical sequelae such as hemorrhagic conversion [128]. Given that shortened treatment windows after stroke onset have been shown to dramatically reduce recruitment rates in stroke trials [129], the possibility of linking AI-tagged findings to clinical trial coordinators offers a potential avenue for screening eligible patients. Moreover, enhanced electronic phenotyping is likely to improve the power of downstream data analyses, as prior work has suggested that the heterogeneous nature of stroke subtypes may contribute to mistaken conclusions from clinical trial data [130].

In neurodegenerative disorders, as well, language processing practitioners have begun to look beyond text data and are taking advantage of the potential for voice to act as an early biomarker [131] of disease that may enhance recruitment. In Alzheimer’s disease (AD), the usage of voice recordings to flag likely cases of AD has been reported using neural networks [132], thus introducing the prospect of identifying potentially afflicted patients without the need for extensive neuropsychological testing [133]. Such efforts build on non-AI-based efforts to recruit patients for AD trials via analysis of vocal features gleaned from mobile applications [134]. Similar studies have been reported in Parkinson’s disease (PD), where machine learning methods have been trained to differentiate PD patients from healthy controls [135, 57]. These methods will require careful planning, including informing participants that their data may result in the detection of potential clinical diagnoses. Subsequently, close integration with clinical care services to provide counseling and adequate treatment to those participants will be required of clinical trial teams, regardless of whether these individuals choose voluntarily to participate in clinical trials.

At the nexus of deep learning and epileptology, work is also being done to adjust enrollment protocols to maximize the chances of success in clinical trials. Work by Romero and Goldenholz has proposed a deep learning model that estimated the contributions of individual patients to a study’s statistical power in epilepsy trials [136]. After simulating placebo and treatment arms with digitally generated cohorts, the authors demonstrated that a neural network could be trained to efficiently compute the “signal to noise ratio” offered by enrolling patients with differing seizure frequencies in randomized trials of a novel antiepileptic agent. The result of this work led to easily interpretable “heatmaps” demonstrating which seizure parameters in newly enrolled patients might maximize the probability of detecting a treatment effect. Notably, their conclusions suggested common patterns of patient characteristics (seizure frequency and variability) that may optimize a trial’s success at the time of enrollment, regardless of the outcome metric used to assess medication response [136]. Even these measures themselves may be rethought with emerging deep learning techniques: the same research group has also shown the ability of a neural network-based scoring system to discriminate drugs from placebo using 21–22% fewer patients than required with the current gold-standard metric for assessing medication response [38].

Moreover, as in other fields, ML is being used to transition from strictly centralized trial designs in neurology as well. Derivation of digital biomarkers of neurologic disease via AI-driven pattern recognition from multimodal data (e.g., wearable devices and sensors) may enable accurate monitoring of patients in neurologic diseases with fluctuating symptomology, such as PD [137, 138], AD [134, 139, 140], and various neuromuscular disorders [141]. The ability to collect such data in an automated fashion may also allow digital biomarkers to avoid many of the imprecisions brought about by basing trial endpoints on subjective behavioral and neuropsychological testing of trial participants [114, 115]. Empatica’s “Embrace2” watch, for instance, is part of a growing list of FDA-approved technologies employing AI as a core feature of its design [142]. The device uses a proprietary ML classifier for seizure monitoring using data from embedded accelerometry and electrodermal activity sensors. The underlying algorithm, which was trained using video EEG labeling by board-certified neurophysiologists surveying > 5000 h of data [143] achieved a sensitivity in prospective trials > 90% for real-time detection of convulsive activity and postictal autonomic dysfunction [144], thus enabling enhanced remote monitoring of patients suffering from seizure disorders. Digital biomarkers based on ML may also help to achieve insights in trials for rare neurologic diseases such as Duchenne muscular dystrophy, where the relative precision of machine-quantified metrics derived from wearable sensors has been suggested as a means of increasing power from small sample sizes and shortening time to endpoint [114]. Additionally, remote monitoring of AI-derived digital biomarkers may elevate patient safety for those who are frail or otherwise unable to be transported directly to clinical trial sites, thus promoting healthier “aging in place” strategies [145] for elderly participants.

In the realm of digital therapeutics, ML may also soon reinvigorate trials that use such technologies as virtual reality (VR) and immersive video games to treat neurologic diseases. Already, there is extensive literature regarding the usage of digital therapeutics in neurology [146], spanning sensorimotor rehabilitation following stroke [147-152] and MS [150], chronic pain [151, 152], depression, and epilepsy management [153]. While the majority of these platforms do not utilize ML as a core feature of their design, potential avenues do exist for its integration. Certain commercial producers of VR for neuropsychiatric applications have begun to integrate AI-driven assistants (i.e., chatbots) into the design of therapeutic video games, helping users with depression to navigate cognitive reframing tasks over the course of their treatment [119]. As interactive language models based on massive “foundation” neural networks evolve (e.g., OpenAI’s ChatGPT platform [154]), the usage of such technologies is slated to increase remarkably in both commercial and research applications over the coming years [105], opening avenues by which to improve the user experience of digital therapeutics in neurology and beyond.

Lastly, given sufficiently large retrospective databases, ML technologies may be trained to recapitulate individual patient outcomes across a range of neurologic conditions, and, once calibrated, they may be used to simulate treatments or forecast progression to select suitable candidates for therapeutic interventions. Neurologic disease often follows highly individualized courses influenced by individual-level and environmental factors, as well as latent disease subtypes that may be unknown at the time of trial enrollment or yet undiscovered [155, 156]. Low success rates in antiepileptic therapy [157], for example, have often been linked to the considerable variability in seizure patterns observed between individual patients. Moreover, such heterogeneity, in combination with well-known placebo effects in epilepsy trials [158, 159] has historically complicated trials of novel antiepileptics [160]. Nevertheless, recent simulation work from Goldenholz and colleagues has exemplified the ability to model approaches to recapitulate complex phenomena such as seizure cycles and clustering from large databases of self-reported seizure data [161]. The deployment of more realistic simulated datasets for longitudinal seizure trajectories may be used in ML-based strategies [136] to identify which study designs and patient characteristics are most likely to yield successful trials. In MS, as well, ML-based digital twins generated through techniques such as representation learning [162] may represent a useful clinical tool to predict disease progression and choice of treatment options given the disease’s relapsing–remitting nature [163, 164]. Notably, in a study reported by the company Unlearn.AI, a neural network trained from subjects enrolled in the placebo arms of 3 MS clinical trials, was able to create a virtual cohort of digital twins that recapitulated longitudinal disease trajectories from the original patient dataset. This work raises the possibility of shortening clinical trial timelines given the ability to quickly and arbitrarily create accurately matched control groups for retrospective cohorts undergoing a variety of experimental MS treatments. The same group has also reported statistical indistinguishability of digital twins created from retrospective MS cohorts [162], suggesting applicability of their approach across many different neurologic disease entities. In addition to simulating control groups, clinical simulations may also be employed to ensure generalizability of trial findings to populations with different demographic compositions. As an example, Chen and colleagues (using a propensity scoring method incorporating the K-nearest neighbors algorithm) recently concluded that rates of serious adverse effects reported in a phase III trial of donepezil would be much higher had the original study been composed of a majority of nonwhite participants [165]. Such conclusions, drawn with the need to organize a physical trial in a separate population, provided useful nuance regarding the drug’s safety profile [166].

## Technical Challenges

Despite its many promises, significant technical, pragmatic, and regulatory hurdles remain before AI technologies become a standard component of clinical trials. The inability of ML models to adequately “explain” their outputs, the potential for AI approaches to fail in prospective validation, and a regulatory environment that must adapt to rapidly evolving developments in computational science pose challenges to implementation.

Interpretability of ML models is of central importance in earning the trust of healthcare providers and clinical trial administrators, who are at the helm of high-stakes patient care. Yet, complex models such as large neural networks often produce outputs (e.g., diagnoses, simulated patients) according to internal mathematical rules that defy the causal, mechanistic explanations that are of highest importance in human reasoning [86]. ML models are often regarded as “black boxes,” [167] whose usage requires leaps of faith that exceed the traditional ethical boundaries of medicine. This does not mean that frameworks for enhanced ML explainability have not begun to emerge. A particularly promising development, for instance, has been the development of “Shapley Additive Explanations” or SHAP values [74, 168]. These metrics, along with alternative explainability metrics developed for the same purpose [169-172], provide a means by which to assess the importance of individual features to a model’s ultimate product. Such an approach may be used to probe ML’s reliance on individual features (e.g., socioeconomic status, race) or even on individual pixels in computer vision tasks [49], thereby contextualizing model predictions in recognizable fashion. Even still, post hoc interpretation typically requires the involvement of a human subject matter expert to verify that a computer’s attributions make mechanistic sense and are free of concerning biases [167]. Solutions to the interpretability gap remain, at least in part, a matter of ethical debate [173]. But from a purely technical perspective, an early solution may involve linking explainability metrics to validated clinical markers. Our group’s previous work in brain MRI, for instance, has shown the ability of various neural network-derived risk scores to closely track the deposition of amyloid plaques and neurofibrillary tangles in AD patients and produce mechanistic “disease process maps” [30, 49]. We note that these results have potential applications in the noninvasive monitoring of drug response in novel trials of AD therapies. Nevertheless, adapting general explainability tools to disease-specific benchmarks defies a one-size-fits-all approach, and implementing these strategies across the full spectrum of human disease—both neurologic and non-neurologic—will require sustained efforts and interdisciplinary collaborations.

There is also the difficulty of implementing AI in clinical trial sites, which requires them to adapt their organizational infrastructure to accommodate the use of ML. At present, the vast majority of published AI models are developed as proofs of principle from retrospective datasets [174], and establishing access to these algorithms requires that clinical support staff receive adequate training in their usage, development, and access to manageable user interfaces (e.g., mobile apps, websites), and integrated into existing operational workflows such as electronic health record systems [175, 176]. Furthermore, even following the organizational and information technology realignments necessary to translate ML models to the point implementation, prospective scrutiny remains a critical factor in ensuring that they are used properly over the course of a clinical trial. Human–computer interactions often differ substantially from a model’s intended usage [177], and regular audits must be performed to ensure that AI implementation is indeed facilitating a clinical trial’s administration rather than hampering it. It is essential that any discordance between preclinical performance and prospective usage (particularly in models developed using synthetic or single-institution datasets) [178] be recognized in real time and that standards for early termination of clinical trials be followed in the case of serious mismatches.

Lastly, regulatory and reporting practices for AI are in flux as governing agencies adapt to a landscape of unprecedented progress. The academic community has begun to develop reporting and protocol development guidelines for clinical trials involving AI [82, 179, 180], thus contributing to a culture of accountability surrounding medical ML among researchers. Moreover, the FDA has moved to define the new category of “Software as Medical Device” (SaMD) and has outlined an updated regulatory approach via its Digital Health Innovation Action Plan [177]. As part of this shift, the agency has outlined a specific Software Precertification Program alongside existing review pathways [177, 181] in order to facilitate streamlined approval of products employing ML in their design. Further work, however, is likely needed in order to ensure consistent quality standards in approvals such as requirements for multi-site algorithm development, dataset auditing, and prospective validation [182]. Conversely, in the EU, uniform pathways for approval of AI-based medical devices have not been developed; instead, accredited “notified bodies” in various member states are given regulatory power to issue “Conformité Éuropéen” (CE [European Conformity]) certifications prior to usage with patients, which are then mutually recognized by member states. The European Parliament, however, has passed the General Data Protection and Regulation (GPDR) law, a stringent set of guidelines that notably requires a strong degree of explainability for algorithms to be deployed in patient care [174]. The requirement to go beyond black-box models is likely to strongly impact the regulatory and innovation environment across the EU for medical AI, despite the lack of a centralized review process.

## Conclusion

As medicine matures in the information age, efforts to derive actionable insights from healthcare data will advance the traditional boundaries of clinical trials. The application of machine learning technologies will require attention to data security as well as privacy and must integrate with the wealth of knowledge found in established medical practice. Responsible development in this arena has the potential to advance the pace of scientific discovery with lasting benefits for patients, clinicians, and society at large.
